# Effects of anabolic steroid use on myocardial perfusion in body-builders: a quantitative cardiovascular magnetic resonance Study

**DOI:** 10.1186/1532-429X-15-S1-P145

**Published:** 2013-01-30

**Authors:** Tevfik F Ismail, Li-Yueh Hsu, Peter J Angell, Andrew Jabbour, Anders M Greve, Carla Gonçalves, Ankur Gulati, Benjamin Hewins, Gillian Smith, Rick Wage, Annette L Dahl, Michael Roughton, Gregory Whyte, Keith George, Dudley J Pennell, Andrew E Arai, Sanjay K Prasad

**Affiliations:** 1 CMR Unit & NHLI Imperial College London, Royal Brompton Hospital & NHLI Imperial College London, London, UK; 2 Laboratory of Cardiac Energetics, National Institutes of Health, Bethesda, MD, USA; 3 Research Institute for Sport and Exercise Sciences, Liverpool John Moore's University, Liverpool, UK

## Background

Anabolic steroids are known to induce left ventricular hypertrophy (LVH). The pathologic LVH due to hypertrophic cardiomyopathy, hypertension, and aortic stenosis has been associated with myocardial perfusion abnormalities. However, the effects of anabolic steroid misuse on myocardial blood flow (MBF) are unknown.

## Methods

Twenty one body-builders were studied - 14 anabolic steroid users and 7 controls matched for age and training history. First pass CMR perfusion imaging was performed on a 1.5T Avanto (Siemens, Erlangen, Germany) after adenosine-induced hyperemia (140 mcg/kg/min) and at rest using a hybrid echo-planar imaging sequence. Images of the base, mid-ventricle and apex were acquired and the myocardium was divided into 16 segments as well as endocardial and epicardial layers. After image registration, a modified Fermi-constrained deconvolution algorithm was applied pixel-wise to quantify absolute MBF. Late gadolinium enhancement (LGE) imaging was performed as well as standard assessment of ventricular volumes, function and LV mass. Data were analysed using a linear mixed effects model.

## Results

Anabolic steroid-using subjects had significant LVH both in terms of maximum wall thickness and indexed LV mass (Table 1). For the whole cohort, endocardial MBF (ml/100g/min) was significantly higher than epicardial MBF at rest (103±29.6 vs 94.5±26.76, p<0.001) but was similar with stress (235 ± 64.8 vs 238 ± 61.8, p=0.363). The difference in resting epicardial versus endocardial resting MBF (β = -13.5, 95% CI: -17.5 to -9.44, p<0.001) was greater with steroid use (β = -23.0, 95% CI: -40.4 to -5.55, p=0.010) than non-use. However, there was also a significant interaction effect with steroid use and the layer examined such that the difference in endocardial MBF between the steroid users and non-users, and the differences between the groups in epicardial MBF was 6.89 ml/100g/min higher (95% CI: 1.93 to 11.8, p=0.006). Resting differences in MBF due to steroid use persisted after adjusting for wall thickness (β = 1.84, 95% CI 0.43 to 3.26, p=0.011 for wall thickness) but were abolished by vasodilator stress. There was no significant difference in myocardial perfusion reserve index (MPI=stress MBF/rest MBF) between the two groups (steroid MPI: 2.49 ± 0.75 vs non-users: 2.45 ± 0.57, p=0.822).

**Table 1:** 

Characteristic - n (%)	Non-users (mean ± SD, n=7)	Steroid users (mean ± SD, n=14)	All patients (mean ± SD, n=21)	P value
Age - years	29.4 (6.4)	29.9 (5.1)	29.7 (5.4)	0.841
Male - n (%)	7 (100)	13 (92.9)	20 (95.2)	0.469
Rest HR - min^-1^	57.9 (4.4)	67.0 (10.8)	64.0 (10.1)	0.047
Stress HR - min^-1^	88.2 (10.3)	92.4 (13.9)	91.1 (12.8)	0.516
Rest mean BP - mmHg	87.0 (14.0)	92.3 (13.5)	90.6 (13.5)	0.415
Stress mean BP - mmHg	87.4 (12.1)	96.8 (17.9)	94.0 (16.6)	0.256
LV-EDV index - ml/m^2^	91.3 (9.9)	95.5 (13.0)	94.1 (12.0)	0.466
LV-ESV index - ml/m^2^	34.3 (5.7)	37.5 (6.9)	36.5 (6.9)	0.306
LV ejection fraction - %	62.4 (3.6)	61.1 (2.7)	61.5 (2.7)	0.343
LV mass index - g/m^2^	79.5 (13.4)	101.8 (14.4)	94.4 (17.5)	0.003
Max wall thickness	9.6 (1.3)	13.2 (2.1)	12.0 (2.5)	<0.001
Late gadolinium enhancement	0 (0)	0 (0)	0 (100)	-

HR=Heart Rate; BP=Blood Pressure; LV-EDV=Left Ventricular End-diastolic volume; LV-ESV=Left Ventricular End-systolic volume; LV=Left Ventricular.

## Conclusions

Hyperemic MBF was similar in strength-trained bodybuilders using anabolic steroids compared with non-users. However, steroid use appeared to exacerbate resting differences in the transmural distribution of perfusion independent of the effects of wall thickness. Further work is required to delineate the mechanisms responsible for these differences in microvascular function.

## Funding

This work is supported by the NIHR Cardiovascular Biomedical Research Unit at the Royal Brompton and Harefield NHS Foundation Trust, and Imperial College. Dr Ismail is supported by the British Heart Foundation. Dr Arai and Dr Hsu are supported by the National Institutes of Health.

